# Supplementary material for: Statistical Modeling and Optimization of Electrospinning for Improved Morphology and Enhanced β-Phase in Polyvinylidene Fluoride Nanofibers
Source: Polymers (Basel). 2023 Nov 7;15(22):4344. doi: 10.3390/polym15224344 (PMC10674670; doi:10.3390/polym15224344)
Supplement: Supplementary file 1 [file polymers-15-04344-s001.zip › polymers-2675691-supplementary.pdf]

Supplementary Data

Table S1: Taguchi Response table for Fiber Diameter.

Response Table for Means

Level	Voltage (kV)	Concentration (wt.%)	Drum Speed (rpm)	Flow rate (ml/hr)
1	125.3	122.7	138.3	122.3
2	135.7	140.3	137.0	142.0
3	147.7	145.7	133.3	144.3
Delta	22.3	23.0	5.0	22.0
Rank	2	1	4	3

Table S2: Taguchi Response table for β-phase Fraction (%).

Response Table for Means

Level	Voltage (kV)	Concentration (wt.%)	Drum Speed (rpm)	Flow rate (ml/hr)
1	67.71	64.34	68.71	71.17
2	68.06	66.93	69.05	67.39
3	69.17	73.67	67.18	66.38
Delta	1.46	9.32	1.86	4.79
Rank	4	1	3	2
